# Crystallographic Analysis and Mimicking of Estradiol Binding: Pedersen et al. Respond

**DOI:** 10.1289/ehp.1307987R

**Published:** 2014-04-01

**Authors:** Lars C. Pedersen, Linda S. Birnbaum, Rajendrakumar A. Gosavi, Gabriel A. Knudsen

**Affiliations:** 1 Laboratory of Structural Biology, National Institute of Environmental Health Sciences, National Institutes of Health, Department of Health and Human Services, Research Triangle Park, North Carolina, USA; 2 Laboratory of Toxicology and Toxicokinetics, National Cancer Institute, National Institutes of Health, Department of Health and Human Services, Research Triangle Park, North Carolina, USA

Previous studies have addressed the biological effects of brominated flame retardants (Birnbaum and Staskal 2004; Koike et al. 2013; Mariussen and Fonnum 2003; Ogunbayo et al. 2008), including a 2-year bioassay study performed by the National Toxicology Program (NTP), which demonstrated that tetrabromobisphenol A (TBBPA) can induce aggressive uterine tumors in rats (NTP 2013). As pointed out by Osimitz et al., TBBPA has been shown to bind poorly to the estrogen receptor, providing the impetus to study other pathways such as disruption of steroid transport and metabolism. Other groups have demonstrated the ability of TBBPA and flame retardant metabolites to inhibit estrogen sulfotransferase (SULT1E1), with IC_50_ (median inhibitory concentration) values near the *K*_m_ for estradiol (Hamers et al. 2008; Kester et al. 2002; Zhang et al. 1998). Our work (Gosavi et al. 2013) was focused solely on understanding the structural mechanism by which these compounds bind to and inhibit SULT1E1’s ability to metabolize estradiol. The results of our work demonstrate that TBBPA and the 3-OH metabolite of BDE-47, although structurally different, bind in a similar manner at the estradiol binding site. This work suggests that these compounds could have an additive effect on the inhibition of this enzyme. We wholeheartedly agree with Osimitz et al. that the results of our work warrant future studies addressing the potential additive effect of these compounds on steroid metabolism in target tissues.
